# Cross-feeding modulates antibiotic tolerance in bacterial communities

**DOI:** 10.1038/s41396-018-0212-z

**Published:** 2018-07-10

**Authors:** Elizabeth M. Adamowicz, Jeffrey Flynn, Ryan C. Hunter, William R. Harcombe

**Affiliations:** 1 Department of Microbiology and Immunology, University of Minnesota, Minneapolis, MN USA; 2 Department of Ecology and Evolutionary Biology, University of Minnesota, St. Paul, MN USA; 3 BioTechnology Institute, University of Minnesota, St. Paul, MN USA

## Abstract

Microbes frequently rely on metabolites excreted by other bacterial species, but little is known about how this cross-feeding influences the effect of antibiotics. We hypothesized that when species rely on each other for essential metabolites, the minimum inhibitory concentration (MIC) for all species will drop to that of the “weakest link”—the species least resistant in monoculture. We tested this hypothesis in an obligate cross-feeding system that was engineered between *Escherichia coli, Salmonella enterica*, and *Methylobacterium extorquens*. The effect of tetracycline and ampicillin were tested on both liquid and solid media. In all cases, resistant species were inhibited at significantly lower antibiotic concentrations in the cross-feeding community than in monoculture or a competitive community. However, deviation from the “weakest link” hypothesis was also observed in cross-feeding communities apparently as result of changes in the timing of growth and cross-protection. Comparable results were also observed in a clinically relevant system involving facultative cross-feeding between *Pseudomonas aeruginosa* and an anaerobic consortium found in the lungs of cystic fibrosis patients. *P. aeruginosa* was inhibited by lower concentrations of ampicillin when cross-feeding than when grown in isolation. These results suggest that cross-feeding significantly alters tolerance to antibiotics in a variety of systems.

## Introduction

Antibiotic-resistant bacteria pose a considerable public health threat worldwide; the World Health Organization reports that 25–50% of hospital-acquired pathogens are now multiple-drug-resistant [1]. Despite extensive research on cellular mechanisms of resistance in many bacterial species [2, 3], a growing body of research suggests that a single-species view of pathogen response to an antibiotic may be incomplete. Many infections are known to involve multiple pathogens [4, 5] or interactions between pathogens and commensals [6–8]. As well, we still have little understanding of how interspecies ecological interactions influence the impact of antibiotics on microbial communities.

Growth in a microbial consortium can influence a species’ antibiotic tolerance by multiple mechanisms [9–11]. Resistant species can protect more sensitive species by degrading antibiotics; for example, production of antibiotic-degrading enzymes by one species causes detoxification of shared growth medium [9, 12, 13]. Additionally, secretions from one species can induce resistance mechanisms in others; for example, by activating stress-response pathways [14] or efflux pump expression [15]. Spatial structure may also play a role in protective interactions; a synthetic community of *Pseudomonas aeruginosa, Pseudomonas protegens*, and *Klebsiella pneumoniae* was found to have greater tobramycin resistance when grown as a multi-species biofilm versus single-species biofilm or multi-species planktonic culture [16]. Less directly, community growth may alter antibiotic resistance by inducing physiological changes in bacteria that increase drug uptake or slow their metabolic rate [17–20]. In many cases, however, mechanisms underlying communities’ effects on resistance remain unclear [5].

Few studies have investigated how exchange of essential nutrients in a bacterial community modulates the impact of antibiotics [21, 22]. When metabolites produced by one organism are used as a nutrient or energy source by another it is known as cross-feeding [23, 24]. This phenomenon is nearly ubiquitous in microbial communities [25–27] and is thought to contribute to our inability to cultivate most bacterial species in isolation [28, 29]. Cross-feeding has also been shown to play a critical role in the human microbiome [30, 32]. Given the ubiquity and importance of cross-feeding in human-associated microbial communities, greater investigation into how cross-feeding influences population and community responses to antibiotics is needed.

Here, we test how cross-feeding changes the effect of antibiotics on bacterial communities. We define tolerance as the ability of species to grow in a given antibiotic concentration. Tolerance as we define it can change as a function of physiological state or environmental conditions, while changes in resistance would require a change in DNA sequence [33]. We hypothesize that when species depend on one another the community tolerance (i.e., the level of antibiotic required to inhibit detectable community growth) will be set by the tolerance of the “weakest link” (the least tolerant community member). Alternatively, community tolerance may be higher than that of the weakest species in monoculture (“community protection” hypothesis), or lower (“community sensitivity” hypothesis). Higher than expected tolerance may occur if one or more species in a community excretes a compound which either actively degrades antibiotics in the medium [9, 34], or which activates tolerance mechanisms such as efflux pump expression in neighboring species [15, 35]. Lower than expected tolerance could result if sublethal concentrations of antibiotic, while not sufficient to arrest or kill any one species, sufficiently disrupt cross-feeding to inhibit community growth.

**Fig. 1:**
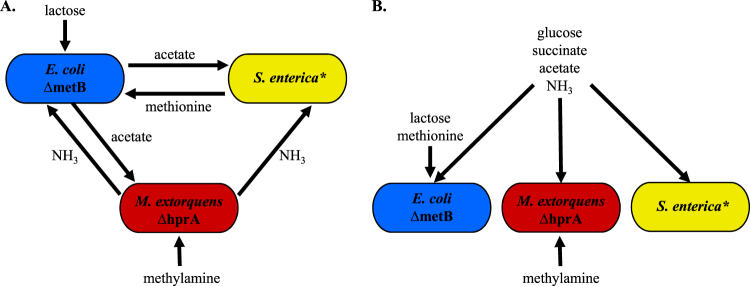
Cooperative and competitive model communities. **a** Cooperative community. Methylamine and lactose are supplied in the growth medium as a nitrogen and a carbon source, respectively. *E. coli* consumes lactose and excretes acetate as a carbon source for *S. enterica* and *M. extorquens*. *S. enterica* secretes methionine for the methionine auxotroph *E. coli*. *M. extorquens*, which has a deletion in *hprA* that renders it unable to assimilate carbon from methylamine, provides nitrogen to the community via methylamine breakdown. **b** Competitive community. Growth medium contains all metabolites necessary for growth of each individual species such that no cross-feeding is necessary to support growth

We tested the impact of cross-feeding using an engineered obligate mutualism involving *Escherichia coli, Salmonella enterica* serovar Typhimurium, and *Methylobacterium extorquens* [36]. In one minimal medium, these species rely on each other for essential metabolites in a cooperative community (Fig. 1a). However, if essential metabolites are provided in the medium, the species can be grown as monocultures, or in a competitive community (Fig. 1b). We compared the tolerance of each species grown in monoculture to tolerance in the mutualism, both overall in the community and at the species level. Our system is ideal to test our “weakest link” hypothesis because the mechanism of dependency between species is known, and the identity of the weakest link can be changed by altering the antibiotic used. This system allows us to rigorously connect observed changes in tolerance to ecological interactions between species.

To test the generality of our hypotheses, and to determine conditions under which deviation from them might occur, we employed multiple experimental setups. We tested two antibiotics with different mechanisms of action—ampicillin is a bacteriocidal inhibitor of cell wall synthesis [9], whereas tetracycline is a bacteriostatic protein synthesis inhibitor [37]. Additionally, ampicillin resistance commonly arises as a function of enzymatic degradation by β-lactamases [37], allowing the potential for cross-protection of less tolerant species [9]. Conversely, tetracycline resistance often involves mutations that would only protect the species that possesses them, such as efflux pump upregulation or target site modification [38]. Tetracycline degradation enzymes do exist, but are far less common than β-lactamases [38, 39]. The impact of these antibiotics was tested in both liquid media and on agar plates to test the influence of spatial structure. Finally, we investigated the effect of cross-feeding on tolerance in a model relevant for cystic fibrosis. This second system involves two components: the pathogen *Pseudomonas aeruginosa*, which grows poorly on mucin, a major carbon source in the cystic fibrosis lung; and a previously defined consortium of anaerobic bacteria that break down mucin into usable metabolites for *P. aeruginosa* [40]. Changing antibiotics, environmental structure, and model systems makes it possible to identify both system-specific and general impacts of cross-feeding on antibiotic tolerance.

Across all treatments and both model systems, resistant bacteria were inhibited by lower concentrations of antibiotic when cross-feeding than when growing independently. However, we found that cross-feeding can conditionally provide protection to less tolerant bacteria. For both ampicillin and tetracycline, cases arose in which tolerance was higher than predicted based on measurements of tolerance in monoculture. Our results demonstrate that metabolic interactions impact antibiotic tolerance in a community and suggest that antibiotic-resistant pathogens may be inhibited by targeting their less tolerant metabolic partners.

## Methods

### Bacterial strains and media

The three-species community contained strains of *Escherichia coli*, *Salmonella enterica*, and *Methylobacterium extorquens* described previously [36]. The *E. coli* str. K12 contains a Δ*metB* mutation. The *S. enterica* strain excretes methionine as a result of mutations in *metA* and *metJ* [41–43]. The *M. extorquens* AM1 Δ*hprA* mutant is unable to assimilate carbon from C1 compounds [44]. In lactose minimal medium, the species rely on each other for essential metabolites. *E. coli* secretes acetate by-products which the other species rely on for a carbon source. *M. extorquens* releases ammonia by-products which provide a source of nitrogen for other species. *S. enterica* secretes methionine, which is essential because our *E. coli* strain is auxotrophic for this amino acid (Fig. 1). Each species has a fluorescent label integrated into its genome: cyan fluorescent protein (CFP) for *E. coli*, yellow fluorescent protein (YFP) for *S. enterica*, and red fluorescent protein (RFP) for *M. extorquens*. Bacteria were grown in minimal Hypho media [36] containing varying amounts and types of carbon and nitrogen, depending on medium type (see Supplementary Table 1).

The cross-feeding system of *Pseudomonas aeruginosa* strain PA14 and a four-species consortium of anaerobic mucin-degrading species has also been previously described. Briefly, *P. aeruginosa* monoculture yield on mucin is relatively low due to its inability to break down mucin into a usable growth substrate. However, when *P. aeruginosa* is co-cultured on mucin with the anaerobic consortium, the latter degrades mucin into usable metabolic by-products and *P aeruginosa* yield increases tenfold [40]. *Pseudomonas aeruginosa* strain PA14 [45] was obtained from D.K. Newman (Caltech). The anaerobic consortium (composed primarily of *Prevotella* sp., *Veillonella* sp., *Fusobacterium* sp., and *Streptococcus* sp.) was derived from human saliva using porcine gastric mucin enrichment as previously described [40].

### Liquid media experiments

Bacteria were inoculated along an antibiotic gradient to measure the minimum inhibitory concentration (MIC) for monocultures and co-cultures. Each species was grown from freezer stocks at 30 °C in species-specific monoculture Hypho medium; monocultures and communities were inoculated from these same monoculture growth conditions (see Supplementary Table 1). Once cultures reached mid-log phase (OD~0.2–0.3), they were diluted 1/200. Cells were inoculated into a 96-well plate, with fresh Hypho and varying concentrations of an antibiotic. The inoculate size for a species was kept constant at ~10^4^ cells per well in monoculture and community (i.e., community treatments started with 3× more total cells than monocultures). Ampicillin was used at 0, 0.2, 0.5, 1, 2, and 5 µg/mL for *E. coli*, *S. enterica*, and the communities, and at 0, 2, 5, 10, 20, and 50 µg/mL for *M. extorquens*; these concentrations provided the best range of sublethal to lethal ampicillin concentrations. Tetracycline was used at 0, 1, 2, 3, 5, and 10 µg/mL for *E. coli*, *M. extorquens*, and the community, and 0, 10, 20, 30, 50, and 100 µg/mL were used for *S. enterica*. 96-well plates were placed into a Tecan InfinitePro 200 at 30 °C for 120 h. Measurements of OD600 were taken every 15 min to track overall bacterial growth, and fluorescence measurements were taken to track growth of individual species. Correlations between colony-forming units (CFU) and OD600 as well as fluorescence can be found in Supplementary Figure 1. MIC was defined as the lowest antibiotic concentration at which no growth (as measured by fluorescence) was seen by 3× the time to detectable growth of the antibiotic-free control.

### Solid media antibiotic susceptibility experiments

Resistance on plates was determined by measuring the zone of inhibition diameter around an antibiotic containing disk. For monocultures, 150 µL of log-phase culture (OD = 0.5) was spread on Hypho plates (1% agar) in a lawn; for communities, 150 µL of culture from each species was mixed, spun down, and re-suspended in 150 µL of the appropriate community medium before plating onto Hypho plates. Discs of filter paper 6 mm in diameter were inoculated with 25 µg antibiotic and left to dry for 10 min. Discs were applied to the center of plates with bacteria, and incubated at 30 °C for 48 h (*E. coli*, *S. enterica*, competitive community) or 72 h (*M. extorquens*, cooperative community), depending on how long it took for cells outside the zone of inhibition to become confluent. Three technical replicates for the zone of clearing were measured for each plate and averaged to provide a single-plate diameter; at least eight biological replicate plates were measured for each condition (see Supplementary Tables 2 and 3 for summary statistics).

### Fluorescence microscopy

Fluorescent images were obtained using a Nikon AZ100 Multizoom macroscope with a C1si Spectral confocal attachment, ×4 objective lens at ×3.40 magnification at the University of Minnesota Imaging Center. 457 nm, 514 nm and 561 nm argon lasers were used to visualize CFP, YFP, and RFP, respectively. Emission maxima are 480 nm for CFP, 550 nm for YFP, and 590 nm for RFP. Disc diffusion Petri plates were placed on the stage and images from 2 × 12 fields of view were obtained and stitched together using Nikon NIS Elements software. Images for each fluorophore were quantified for fluorescence location and overlaid using Fiji image analysis software [46] (see Supplementary Tables 4 and 5 for summary statistics).

### Testing β-lactamase production

Nitrocefin discs (Sigma-Aldrich, 49862) were used to determine if *M. extorquens* was producing a β-lactamase. For solid medium, cells were scraped off agar and suspended in the appropriate liquid medium; the OD600 of the suspension was then diluted to ~0.5, to match the OD600 of liquid cultures. Discs were placed on a microscope slide and 15 µL of liquid culture or diluted solid medium suspension was added to the disc. After 60 min, a color change from yellow to purple/pink indicated the production of a β-lactamase that hydrolysed the nitrocefin in the disc [47]. As a positive control, an *E. coli* strain carrying a pBR322 plasmid, which contains a *bla* β-lactamase gene was also tested. Plasmid-free *E. coli* were used as a negative control.

### *Pseudomonas aeruginosa* cross-feeding model

Antibiotic tolerance assays were performed in minimal medium containing 1 mM magnesium sulfate, 60 mM potassium phosphate (pH 7.4), 90 mM sodium chloride, trace minerals [48] and supplemented with autoclaved and dialyzed pig gastric mucin (30 g/L, Sigma-Aldrich) for co-cultures; mucin or glucose (12 mM) was used for *P*. *aeruginosa* monoculture as indicated. Ampicillin was added at indicated concentrations. For mucin-fermenting community assays, cultures were inoculated from freezer stock characterized previously [40] and allowed to grow under anaerobic conditions containing carbon dioxide, hydrogen and nitrogen (5:5:90) at 37 °C for 48 h. For *P*. *aeruginosa* assays, cultures were inoculated from overnight cultures grown in LB and grown aerobically while shaking at 37 °C for 16 h. Optical densities were determined using a Biotek Synergy H1 plate reader, and are given as mean and standard deviation of three replicates.

Cross-feeding assays were performed as described previously [40]. Briefly, a mucin-fermenting community from freezer stock was inoculated into the minimal mucin medium and allowed to grow for 48 h anaerobically at 37 °C. This culture (OD ≈ 0.8) was used to inoculate the lower phase (1:100 dilution) which contained 2 mL of minimal mucin medium, 1% agar, and supplemented with ampicillin as indicated. After solidification of the mucin-fermenting agar cultures in 16 mm glass culture tubes, *P. aeruginosa* PA14 was added to buffered media containing no mucin and 0.7% agar to 1/1000 from an LB overnight culture (inoculum CFU/mL ~5 × 10^7^). This mixture was then added to the top of the mucin-fermenting community and allowed to solidify. This allowed oxygen to diffuse to *P. aeruginosa* from the top of the tube and mucin degradation products to diffuse from the anaerobic community below. After 60 h at 37 °C, the top agar section (containing PA14) was removed, homogenized by pipette in sterile saline, serially diluted, and plated on LB agar to enumerate PA14.

### Statistical analyses

For liquid and solid media assays, at least eight biological replicates of each treatment were obtained for each antibiotic. Pairwise comparisons between monocultures and co-cultures were conducted using a Mann–Whitney *U* test with an applied Bonferroni correction for ten multiple comparisons. For *P. aeruginosa* cross-feeding assays, triplicate experiments were performed for each antibiotic concentration and community type. Normalized CFU values were calculated by dividing CFU at each antibiotic concentration by the CFU at 0 µg/mL ampicillin. Comparisons of normalized values at each concentration were performed using a Mann–Whitney *U* test. Raw data for these experiments is found in Supplementary Figure 8.

## Results

### Obligate cross-feeding in the cooperative community reduces the amount of antibiotic necessary to inhibit resistant bacteria in liquid media

The MIC of each species was tested in monoculture, cooperative community (cross-feeding), and competitive community. MIC was defined as the lowest concentration of antibiotic at which no growth (as measured by species-specific fluorescent markers) was detected after 3× the time to detection of growth in the relevant antibiotic-free control. This metric was used to take into account different growth rates of each of our species, and each growth condition. Media compositions and carbon sources for each growth condition can be found in Supplementary Table 1.

**Fig. 2:**
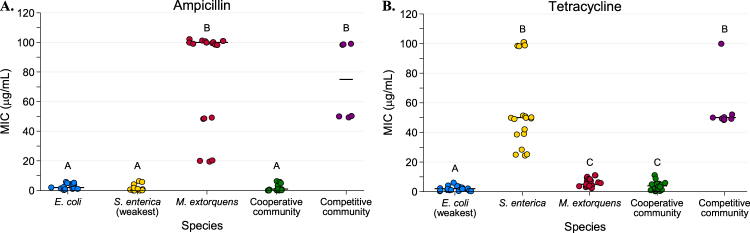
Minimum inhibitory concentration (MIC) values for each monoculture and community type in ampicillin (**a**) and tetracycline (**b**) based on total population OD600. The “weakest link” species (i.e., the species with the lowest median MIC in monoculture) is indicated on the *x*-axis. Bars represent median values. MIC is defined as the minimum concentration of antibiotic required to inhibit growth by three times the time to observable growth of the antibiotic-free control. Cultures were grown on a Tecan plate reader with measurements every 15 min. At least eight replicates were performed for each species/antibiotic combination. Pairwise comparisons of median MIC were performed using a Mann–Whitney *U* test, with a Bonferroni correction applied for ten pairwise multiple comparisons. Shared letters indicate non-significant differences between groups

In monoculture experiments species’ MICs varied widely from each other. When grown in the presence of ampicillin, *M. extorquens* had a median MIC of 100 μg/mL, which was significantly higher than the 2 and 1 μg/mL necessary to inhibit *E. coli* and *S. enterica*, respectively (Fig. 2a, *P* < 0.0001 for each). In tetracycline, *S. enterica* had a median MIC of 50 μg/mL (Fig. 2b**)**. This was significantly higher than the median MIC of 5 μg/mL in *M. extorquens* (*P* < 0.0001) and 2 μg/mL in *E. coli* (*P* < 0.0001). We note that the spread in MIC for the most tolerant species is in part due to increasing step-size between antibiotic concentrations along the gradient; for example, growth at 50 vs. 100 μg/mL represents a single step, as does 5 vs. 10 μg/mL.

Consistent with the weakest link hypothesis, antibiotic concentrations needed to inhibit resistant species decreased substantially when bacteria were grown in an obligate mutualism rather than in monocultures. Fifty-fold less ampicillin was needed to inhibit the cooperative community (1 μg/mL) than to inhibit *M. extorquens* in monoculture (Fig. 2a, *P* < 0.0001). Similarly, the median MIC of tetracycline for *S*. *enterica* decreased significantly from 50 μg/mL in monoculture to 4 μg/mL in the cooperative community (Fig. 2b, *P* < 0.0001).

**Fig. 3:**
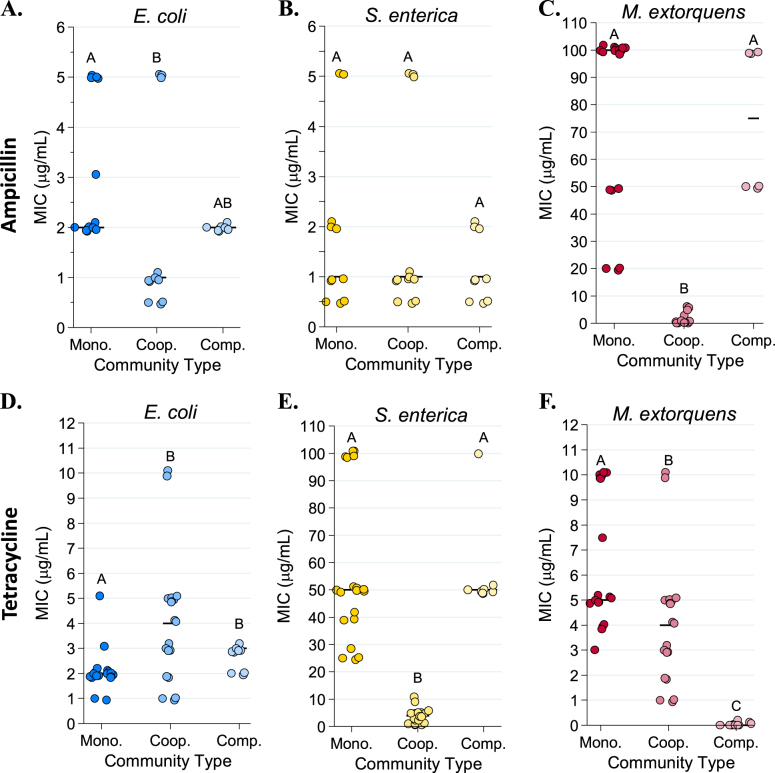
Species-specific minimum inhibitory concentration (MIC) values in monoculture, cooperative community, and competitive community in ampicillin (**a**–**c**) and tetracycline (**d**–**f**). MIC was defined as the minimum concentration of antibiotic required to inhibit growth by three times the time to detectable growth of the antibiotic-free control MICs were calculated based on fluorescence (CFP for *E*. coli, YFP for *S. enterica*, and RFP for *M. extorquens*) recorded on a Tecan plate reader with fluorescence measurements every 15 min. Pairwise comparisons of median MIC were performed using a Mann–Whitney *U* test, with a Bonferroni correction applied for three multiple comparisons. Shared letters indicate non-significant differences between clusters

We next distinguished the effect of species interactions from community complexity by measuring the MIC of the bacterial community when species were competing for common resources (Figs. 1b and 2). The median MIC for resistant bacteria was not significantly different in the competitive community than it was in monoculture (for *M. extorquens* in ampicillin Fig. 3c, *P* > 0.90; for *S. enterica* in tetracycline, Fig. 3e, *P* = 0.80). Therefore, decreased tolerance of resistant species to these antibiotics was a result of metabolic interdependence, rather than simply the presence of other species.

As an additional control, we examined the monoculture and competitive co-culture MIC of the three species using carbon sources that matched those that they consume in cooperative community (Supplementary Figure 2). We found that the same qualitative patterns were observed, with *M. extorquens* showing high tolerance to ampicillin and *S. enterica* demonstrating high tolerance to tetracycline in both monoculture and competitive community.

### Tolerance to tetracycline was higher than expected in community

Unexpectedly, we saw a small but significant trend towards protection of *E. coli* in cooperative community in tetracycline. *E. coli* median monoculture MIC was 2 µg/mL while median MIC in the cooperative community was 4 μg/mL (*P* = 0.0111, Fig. 3d); we expected these MICs to match, as *E. coli* was the weakest link in tetracycline. This effect was not due to higher starting cell density in community, which can increase antibiotic tolerance [49] (Supplementary Figure 3A). As tetracycline is known to rapidly photolyse [50], we predicted that its increased time to detectable growth in the cooperative community might be protecting *E. coli* by allowing time for tetracycline to break down. Several lines of evidence indicate that tetracycline breakdown is occurring as *E. coli* experiences a delay in nutrient access when obtaining metabolites from cross-feeding partners instead of growth medium. First, *E. coli* monoculture MIC increases if tetracycline-containing media sits for 20 h before cells are added (Supplementary Figure 3A). Second, the time to detectable *E. coli* growth is significantly longer in cooperative community than in monoculture (Supplementary Figure 3B, *P* < 0.001). Third, the MIC of *E. coli* in monoculture increased if cells sat in tetracycline for 20 h before nitrogen and methionine were added to the media (*P* = 0.001, Supplementary Figure 3A).

### Interspecies competition sets *M. extorquens* tolerance in tetracycline

*M. extorquens* growth in tetracycline also did not follow the hypothesized weakest link pattern, though for different reasons than *E. coli*. In monoculture, tetracycline MIC for *M. extorquens* was 5 μg/mL, while in the competitive community *M. extorquens* growth was not observed even in the absence of antibiotic (Fig. 3f). *M. extorquens* was able to grow in the ampicillin experiments because it can grow at high ampicillin concentrations where its better competitors are antibiotic-inhibited (Supplementary Figure 4). This suggests that growth patterns of *M. extorquens* in tetracycline are governed by competitive ability rather than resistance, while in ampicillin *M. extorquens* experiences competitive release (where removal of a stronger competitor species, in this case by antibiotic, allows a weaker competitor to grow).

### Resistant bacteria are also constrained by sensitive partners in structured environments

We next tested whether growth on agar (rather than growth in liquid media) altered the impact of species interactions on antibiotic resistance. We hypothesized that spatial structure might enhance the ability of resistant bacteria to protect metabolic partners from degradable antibiotics like ampicillin, and thereby eliminate reduction of tolerance in the cross-feeding system.

**Fig. 4:**
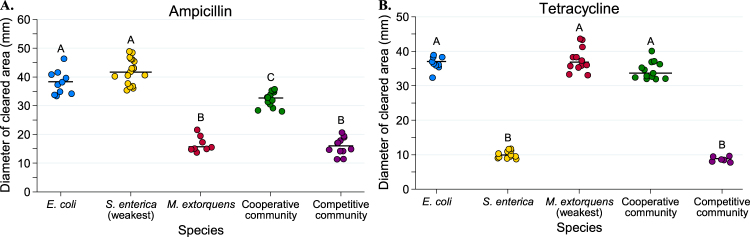
Diameters of zones of clearing for ampicillin (**a**) and tetracycline (**b**) disc diffusion assays. The diameter of the zone of clearing was measured three times for each plate and averaged for a single-plate measurement. At least eight replicate plates were measured for each monoculture and community type. The “weakest link” species (i.e., the species with the largest median zone of clearing in monoculture) is indicated on the *x*-axis. Pairwise comparisons of the zone of clearing for each monoculture and community was performed with a Mann–Whitney *U* test with Bonferroni adjustment for multiple comparisons. Significant differences are noted by different letters above each cluster; shared letters represent non-significant differences

**Fig. 5:**
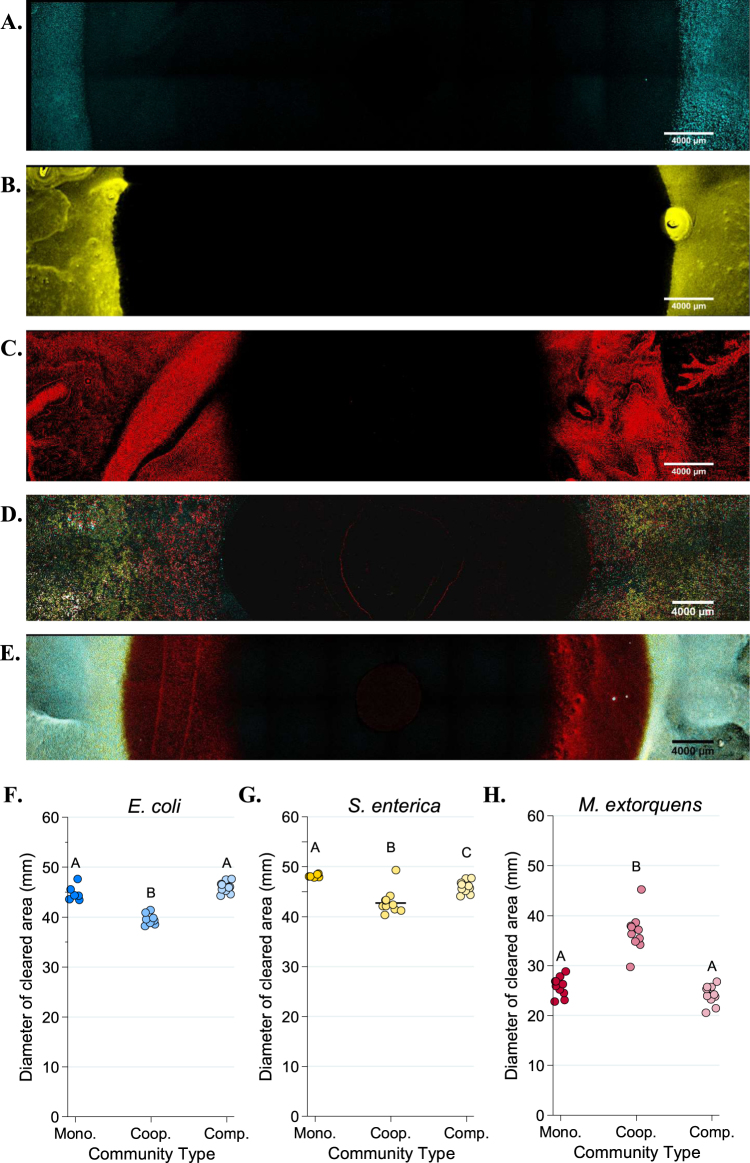
Fluorescent microscopy images of Petri plates with ampicillin antibiotic discs. An AZ100 confocal fluorescent macroscope at ×3.40 magnification was used to image 12 × 2 fields of view of each Petri plate to visualize *E. coli* (CFP, in blue), *S. enterica* (YFP, in yellow) or *M. extorquens* (RFP, red). **a**–**e** Representative images of *E. coli* monoculture (**a**), *S. enterica* monoculture (**b**), *M. extorquens* monoculture (**c**), cooperative community (**d**), and competitive community (**e**). Quantification of the diameter of the species-specific zone of clearing for *E. coli* (**f**), *S. enterica* (**g**), and *M. extorquens* (**h**) in each growth condition was performed in Elements software. The average of three technical replicate diameters was calculated to obtain a single measurement. At least 6 biological replicates were obtained for each species/growth condition. Pairwise comparisons of median diameter of clearing were performed using a Mann–Whitney *U* test, with a Bonferroni correction applied for three multiple comparisons. Significant differences are noted by different letters above each cluster

**Fig. 6:**
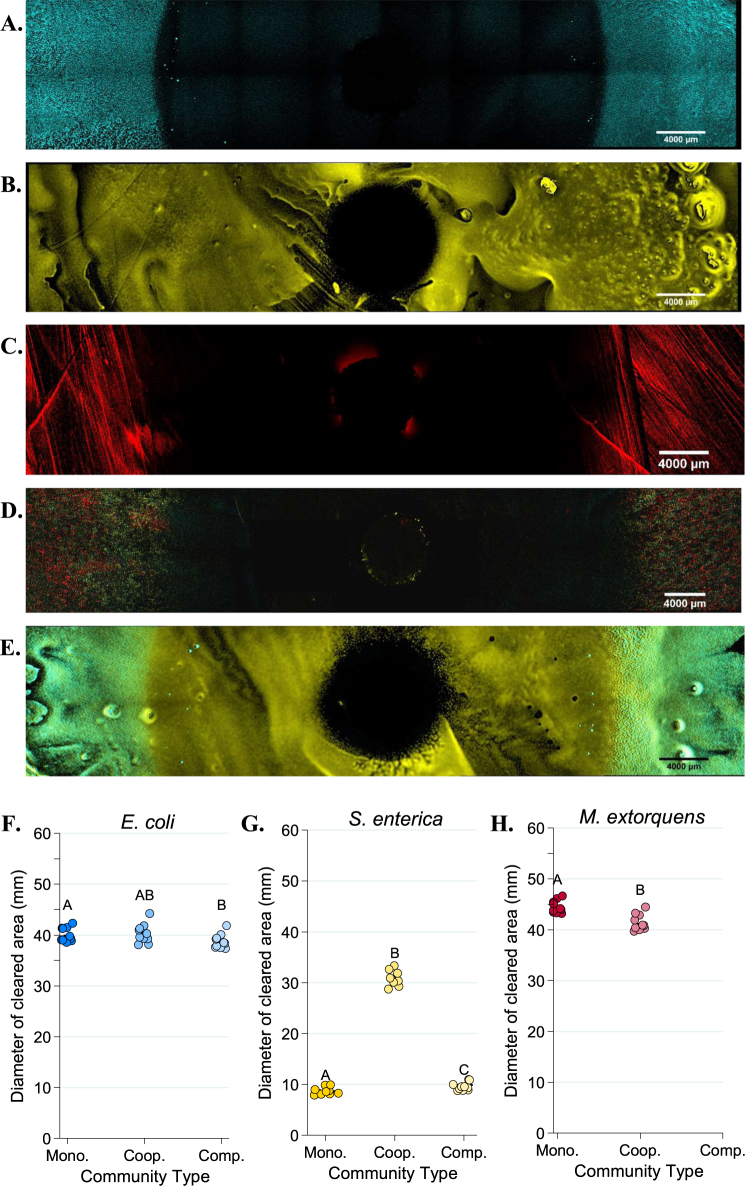
Fluorescent microscopy images of Petri plates with tetracycline antibiotic discs. An AZ100 confocal fluorescent macroscope at ×3.40 magnification was used to image 12 × 2 fields of view of each Petri plate to visualize *E. coli* (CFP, in blue), *S. enterica* (YFP, in yellow) or *M. extorquens* (RFP, red). **a**–**e** Representative images of *E. coli* monoculture (**a**), *S. enterica* monoculture (**b**), *M. extorquens* monoculture (**c**), cooperative community (**d**), and competitive community (**e**). Quantification of the diameter of the species-specific zone of clearing for *E. coli* (**f**), *S. enterica* (**g**), and *M. extorquens* (**h**) in each growth condition was performed in Elements software. The average of three technical replicate diameters was calculated to obtain a single measurement. At least 6 biological replicates were obtained for each species/growth condition, except for *M. extorquens* in competition in tetracycline, for which no RFP signal could be detected. Pairwise comparisons of median diameter of clearing were performed using a Mann–Whitney *U* test, with a Bonferroni correction applied for three multiple comparisons. Significant differences are noted by different letters above each cluster

Tolerance patterns on agar largely mirrored results from liquid media. Less antibiotic was needed to inhibit resistant bacteria in cooperative community than was needed in monoculture (Figs. 4–6). Note that small clearing diameters signify high resistance, so relative rankings in Figs. 4–6 are the inverse of Figs. 2 and 3. Ampicillin cleared growth of *M. extorquens* out to a median diameter of 37.2 mm in cooperative community, but only 26.55 mm in monoculture (Fig. 5h, *P* = 0.0006). Similarly, on tetracycline, *S. enterica* had a median clearing diameter of 8.67 mm in monoculture and 41.2 mm in the cooperative community (Fig. 6g, *P* = 0.0012). In the competitive community, zones of clearing matched those of the most resistant monoculture (Fig. 4a, *M. extorquens* vs. competitive community *P* *=* 0.999; Fig. 4b, *S. enterica* vs. competitive community *P* = 0.4242). As in liquid, *M. extorquens* is only observed in ampicillin competitive community at diameters where it has higher tolerance than *E. coli* and *S. enterica* (Fig. 5e), and not at all in in tetracycline competitive community (Fig. 6e, h). Finally, using acetate as the carbon source for *S. enterica* and *M. extorquens* in monocultures and competitive community again showed qualitatively similar results (Supplementary Figure 5).

Though *M. extorquens* had lower tolerance for ampicillin in cooperative community, we did observe cross-protection of more sensitive species. The cooperative community overall had a significantly smaller zone of inhibition than either *S. enterica* or *E. coli* monocultures (Fig. 4a, *P* < 0.0001 for *S. enterica* and *P* = 0.01 for *E. coli*). Quantification of fluorescence on these plates indicates that inhibition of both sensitive species was reduced in cooperative community (Fig. 5f, *E. coli* monoculture vs. cooperative community diameter *P* = 0.0057, and Fig. 5g, *S. enterica* monoculture vs. cooperative community diameter *P* = 0.02). This can also be observed qualitatively (Fig. 5a and b vs. d). We found that protection was not due to an increase in initial cell density on community versus monoculture plates (Supplementary Figure 6A), and that *M. extorquens* was responsible for providing protection (Supplementary Figure 6B). Consistent with the observed cross-protection, genes encoding ampicillin degrading β-lactamases were found in the genome of *M. extorquens*. Nitrocefin disks were used to demonstrate β-lactamase activity when *M. extorquens* was grown on agar in the presence of ampicillin (Supplementary Figure 7).

### Metabolic dependency reduces antibiotic tolerance of a pathogen

To test whether our findings extend to a medically relevant system, we investigated how co-culturing influences the effective tolerance of *Pseudomonas aeruginosa* to ampicillin*. P. aeruginosa* is a leading cause of morbidity and mortality in people with cystic fibrosis [51]. It was recently demonstrated that *P. aeruginosa* can cross-feed on carbon generated by mucin-degrading anaerobes that are also associated with CF lung disease [40]. In addition to its medical relevance, this system is distinct from our previous system in that cross-feeding is not obligate (*P. aeruginosa* growth on mucin decreases but is not abolished by the absence of anaerobes). We tested how ampicillin influenced the growth of *P. aeruginosa*, when grown alone on mucin versus in a facultative cross-feeding co-culture.

**Fig. 7:**
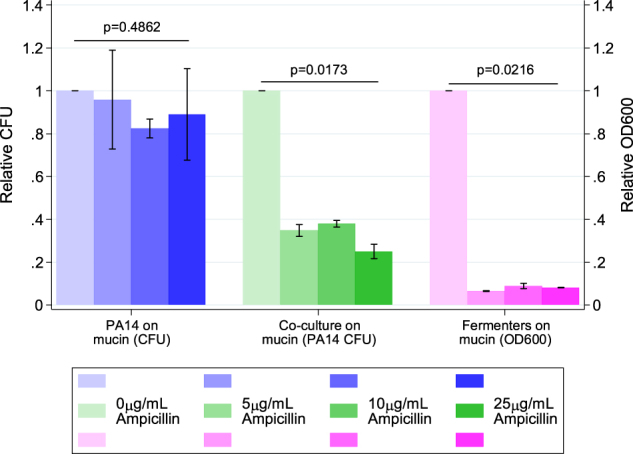
Ampicillin tolerance of *Pseudomonas aeruginosa* PA14 grown, PA14 cross-feeding with a mucin-fermenting community, and the mucin-fermenting community alone. PA14 colony-forming units (CFUs) from monocultures and co-cultures were enumerated by plating cells from each ampicillin concentration on LB agar after 16 h of growth. Fermenter community OD600 was measured with a Biotek Synergy H1 plate reader after 48 h of growth. Normalized OD600 and CFU values were calculated for each concentration of ampicillin by dividing raw values for the OD600 or CFU value at that concentration by the raw OD600 or CFU value of growth at 0 µg/mL ampicillin. Each point represents the mean and standard deviation of triplicate samples. *P*-values were calculated using a Kruskal–Wallis test across ampicillin concentrations using relative CFU (for PA14) or OD600 (for fermenters)

Consistent with previous findings [52], *P. aeruginosa* was highly resistant to ampicillin in monoculture (Fig. 7, Supplementary Figure 8A). No observable decrease in final CFU count was observed across any concentration out to 25 μg/mL of the drug. In contrast, ampicillin inhibited the final density of mucin-degrading anaerobes (as measured by OD600), starting at 5 μg/mL (*P* *=* 0.0216) (Fig. 7, Supplementary Figure 8B). Consistent with expectations, ampicillin also reduced the final CFU of *P. aeruginosa* grown in co-cultures on mucin, starting at 5 µg/mL ampicillin (*P* = 0.0173). These data suggest that applying antibiotic to inhibit the growth of cross-feeding partners can inhibit resistant species even in a non-obligate cross-feeding system of clinical significance.

## Discussion

Our results demonstrate that metabolic dependency between microbial community members plays a critical role in mediating the effect of antibiotics. In both mass-action (liquid) and structured (solid) environments, we observed that bacterial species that show high levels of tolerance to a given antibiotic in monoculture are inhibited at much lower concentrations in an obligate mutualism. The constraint of cross-feeding on bacterial tolerance was consistent across drugs and microbial systems; this was true for two antibiotics with different modes of action and extended to a medically relevant system with facultative cross-feeding. *P. aeruginosa* growth was reduced by substantially lower concentrations of ampicillin when the pathogen was cross-feeding off of mucin-degrading anaerobes that were sensitive to the drug.

We have shown that the ability of a given bacterial species to grow in the presence of an antibiotic is a combination of its intrinsic tolerance and the tolerance of species on which it relies for metabolites. Dependence on other bacteria reduced the MIC of bacteria with high resistance in monoculture, regardless of the antibiotic and mechanism of action. This change in MIC was driven by inhibition of a beneficial partner rather than a change in the resistance of the focal species. The effective tolerance of a cross-feeding network, therefore, is generally set by the “weakest link” species; that is, the species with the lowest resistance to the antibiotic, whose tolerance in community usually matches its monoculture tolerance. This suggests that antibiotics will often be more effective at controlling microbial communities where there is extensive metabolic interdependence, and that tolerance in cross-feeding communities can be approximated from monoculture studies.

Unexpectedly, we did see deviations from our weakest link hypothesis. *E. coli* had a higher tetracycline MIC in the cross-feeding community than in monoculture, suggesting some protective effect of cooperative community growth. This slight, but significant, increase was likely driven by an increase in time to detectable *E. coli* growth when cross-feeding. Tetracycline breaks down rapidly, so this delay likely allowed *E. coli* to experience reduced antibiotic concentrations. Similar antibiotic dynamics may often occur in clinical or environmental settings [50, 53], where metabolically inactive “persisters” commonly survive antibiotic treatment by delaying growth [33], particularly in the case of bacteriostatic drugs such as tetracycline. Although we have evidence that a delay in growth in cooperative community coupled with tetracycline breakdown can explain the increased *E. coli* tolerance to tetracycline, it is important to note that other factors may also contribute.

Community context further altered tolerance by enabling cross-protection of less tolerant species by more tolerant partners. On agar with ampicillin, both *E. coli* and *S. enterica* grew closer to the antibiotic disc in the presence of *M. extorquens*. This protection was likely caused by degradation of the antibiotic due to β-lactamase activity in *M. extorquens*. Our results are consistent with previous observations that spatial structure can allow bacteria to lower local antibiotic gradients sufficiently to permit growth of sensitive isolates [9]. There were limits on the extent of cross-protection in our community, however. The cross-feeding community increased tolerance of *E. coli* and *S. enterica* but, tolerance of *M. extorquens* was still lower in the cooperative community than it was in monoculture. Cross-protection may reduce the magnitude of the constraints placed on resistant species by their more sensitive metabolic partners, but it does not eliminate this constraint. As well, degradative enzymes are not available for all antibiotics nor for all bacterial species, limiting the ubiquity of this mechanism. Further research is needed on the interaction of cross-protection and cross-feeding, particularly in polymicrobial infection contexts, as these studies may help direct antibiotic choice.

This study also demonstrates some issues which can arise when measuring the effect of antibiotics in microbial communities. It has previously been shown that MIC is a problematic metric that can be influenced by factors such as changes in initial microbial density, or metabolic state [33, 49]. In our study, it was not possible to measure the tolerance of *M. extorquens* to tetracycline in the competitive community, as *M. extorquens* was always outcompeted. The competitive release of *M. extorquens* in ampicillin-treated competitive communities again deviates from standard patterns for MIC. This may also impact antibiotic choice in polymicrobial infections. If, for example, a pathogen grown in monoculture is highly antibiotic-resistant but limited in vivo by less tolerant competitors, application of high levels of antibiotic might only serve to remove competitor species that would have otherwise kept the pathogen at bay; this has been observed in *C. difficile* infections, which are often precipitated by antibiotic-mediated depletion of healthy intestinal microbiome species [54–56]. Our results highlight that the community context further complicates challenges associated with interpreting MIC measurements.

The constraint of cross-feeding on antibiotic tolerance also extended to a microbial community relevant to cystic fibrosis. It should be noted that this system involved facultative cross-feeding, so inhibiting anaerobes only reduced the yield of *P. aeruginosa* by twofold. While this reduction is substantially smaller than the complete elimination of growth in the obligate system, twofold changes may be medically relevant [57–61]. More broadly, the constraint in this treatment speaks to the generality of our findings. Even in scenarios with less extreme metabolic dependency, the impact of antibiotics can be magnified when highly resistant species are cross-feeding from less resistant species. Given that metabolic interactions are common in infection contexts [6, 7], this work suggests that even narrow-spectrum antibiotics, designed to target a single species, may have widespread effects throughout a metabolically interconnected community.

Our results highlight that mutualistic networks are highly susceptible to environmental change. This result is consistent with work in other ecological systems from plant-pollinator to insect-symbiont [62–64]. Integrating these ecological concepts into a microbial perspective may allow greater precision in our medical practices. Broad-spectrum reductions of bacteria in the gut can cause long-lasting negative health outcomes such as facilitating infections by *Clostridium difficile* [56]. To develop precision treatments, predicting the impact of a drug on a focal population, and how a drug will affect off-target members of a microbial community, is essential. Our work highlights that precision microbiome management will require not only improved pharmacology but also a more comprehensive understanding of ecological interactions in microbial systems [65].

## Electronic supplementary material

Supplemental material
