# Supplementary material for: Cross-feeding modulates antibiotic tolerance in bacterial communities
Source: ISME J. 2018 Jul 10;12(11):2723–35. doi: 10.1038/s41396-018-0212-z (PMC6194032; doi:10.1038/s41396-018-0212-z)
Supplement: Supplementary file 1 — Supplemental material [file 41396_2018_212_MOESM1_ESM.pdf]

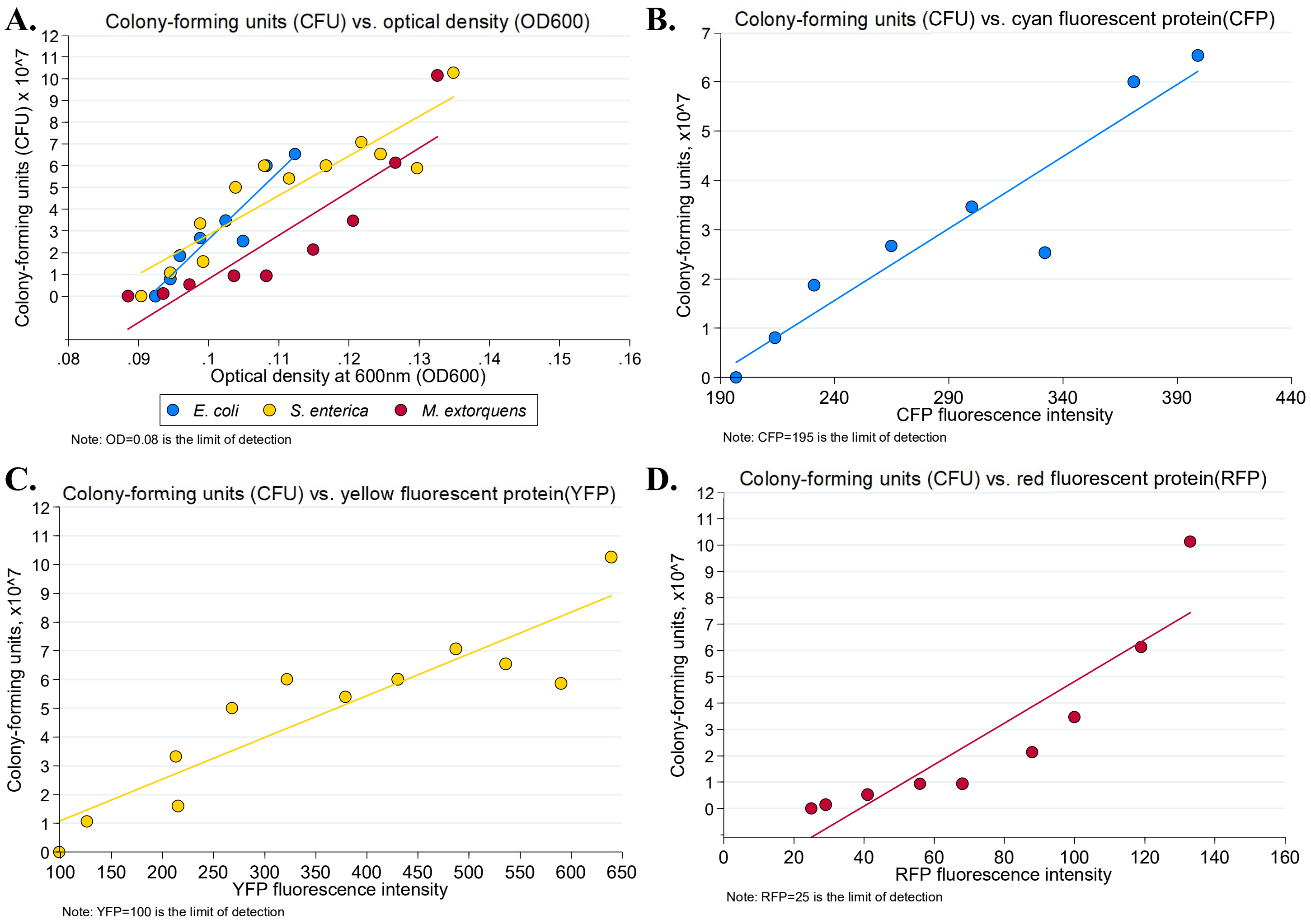

Supplementary figure 1. Correlations between colony-forming units (CFU) and optical density (OD600) for each of *E. coli*, *S. enterica*, and *M. extorquens* (**A**), between CFU and cyan fluorescent protein (**B**), between CFU and yellow fluorescent protein (**C**), and between CFU and red fluorescent protein (**D**). Briefly, cultures of each species were grown to mid-log phase at 30°C (OD600~0.2), diluted twofold eleven times, and the dilutions measured for their OD600 and fluorescence in a Tecan plate reader. Each dilution was then plated for CFU and the resulting CFU/mL values were calculated and plotted against measured OD600 and fluorescent protein values.

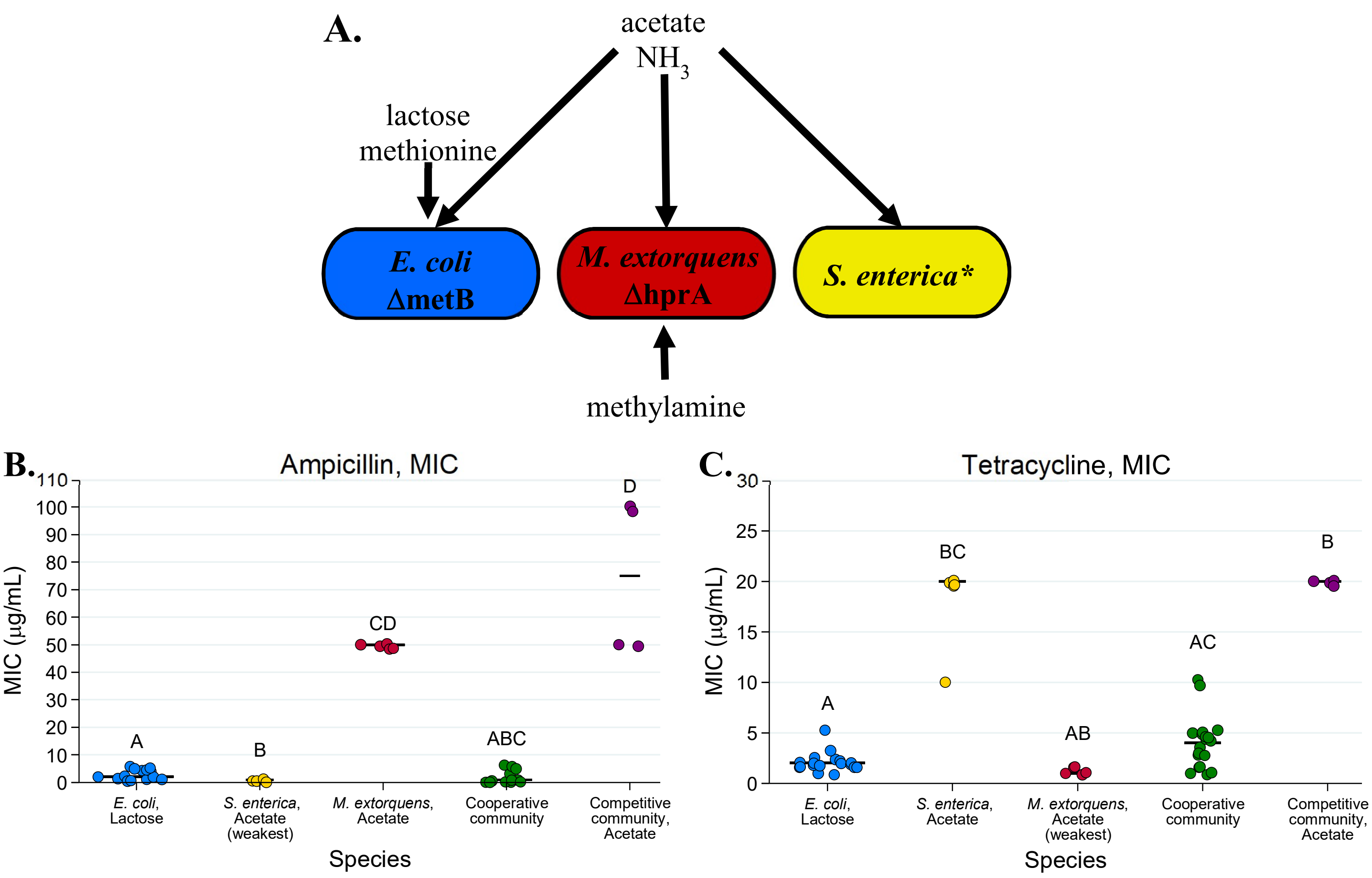

Supplementary figure 2. Acetate as the sole carbon source for *M. extorquens* and *S. enterica* in monoculture and competitive community. **A.** Competitively grown three-species community using acetate. Growth medium contains all metabolites necessary for the growth of each individual species, with the carbon sources provided matching that of the carbon sources available to each species in cooperative community via cross-feeding. Minimum inhibitory concentrations (MICs) in for ampicillin (**B.**) and tetracycline (**C.**) were calculated as previously described. At least four replicates were performed for each community type. Matching letters represent statistical non-significance in medians between groups.

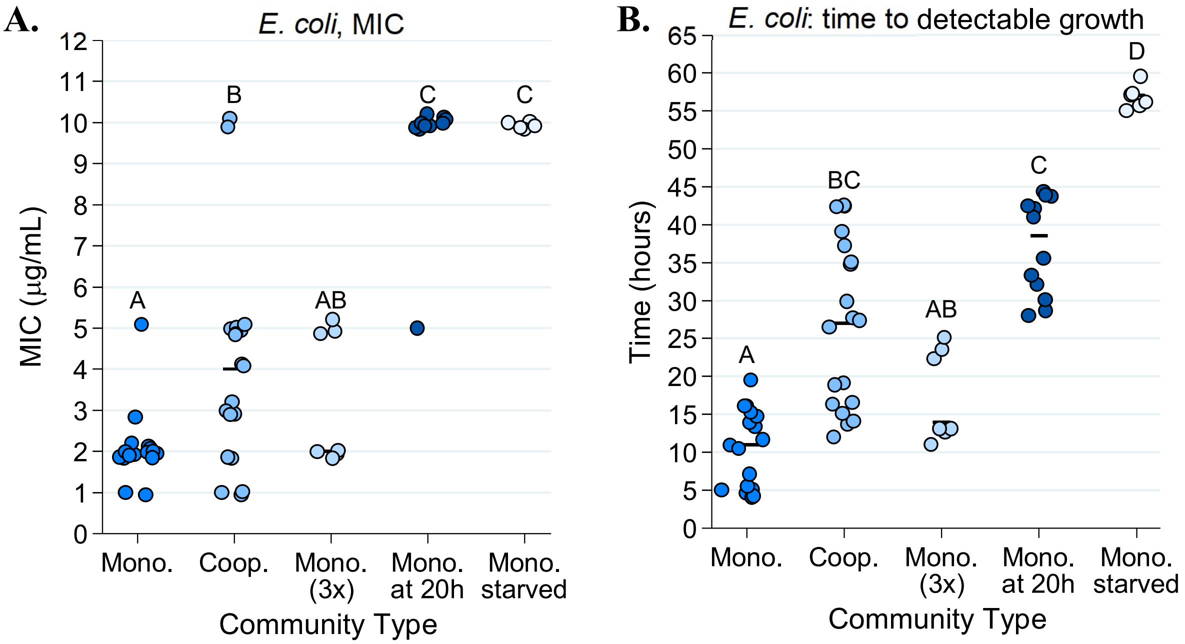

Supplementary figure 3. **A.** Minimum inhibitory concentrations (MICs) of tetracycline for *E. coli* in different growth conditions. MIC was calculated as previously described. 3x mono is monoculture *E. coli* at three times the starting density of conventional monocultures. Mono. at 20h is sterile *E. coli* monoculture medium containing antibiotic and incubated for 20 hours prior to *E. coli* inoculation. Mono. starved is *E. coli* monocultures in which nitrogen and methionine are added 20 hours post-*E. coli* inoculation. **B.** Time to detectable growth of *E. coli* in different growth conditions. Cyan fluorescent protein (CFP) was used to monitor the growth of *E. coli.* Time to detectable growth was recorded as the first time point in which CFP above background levels was detected. In the Mono. at 20h and Mono. starved data, the 20 hour incubation period is included in the time to detectable growth. Pairwise MIC comparisons were performed using a Mann-Whitney U test with Bonferroni adjustment for ten multiple comparisons. Shared letters represent nonsignificant differences between groups.

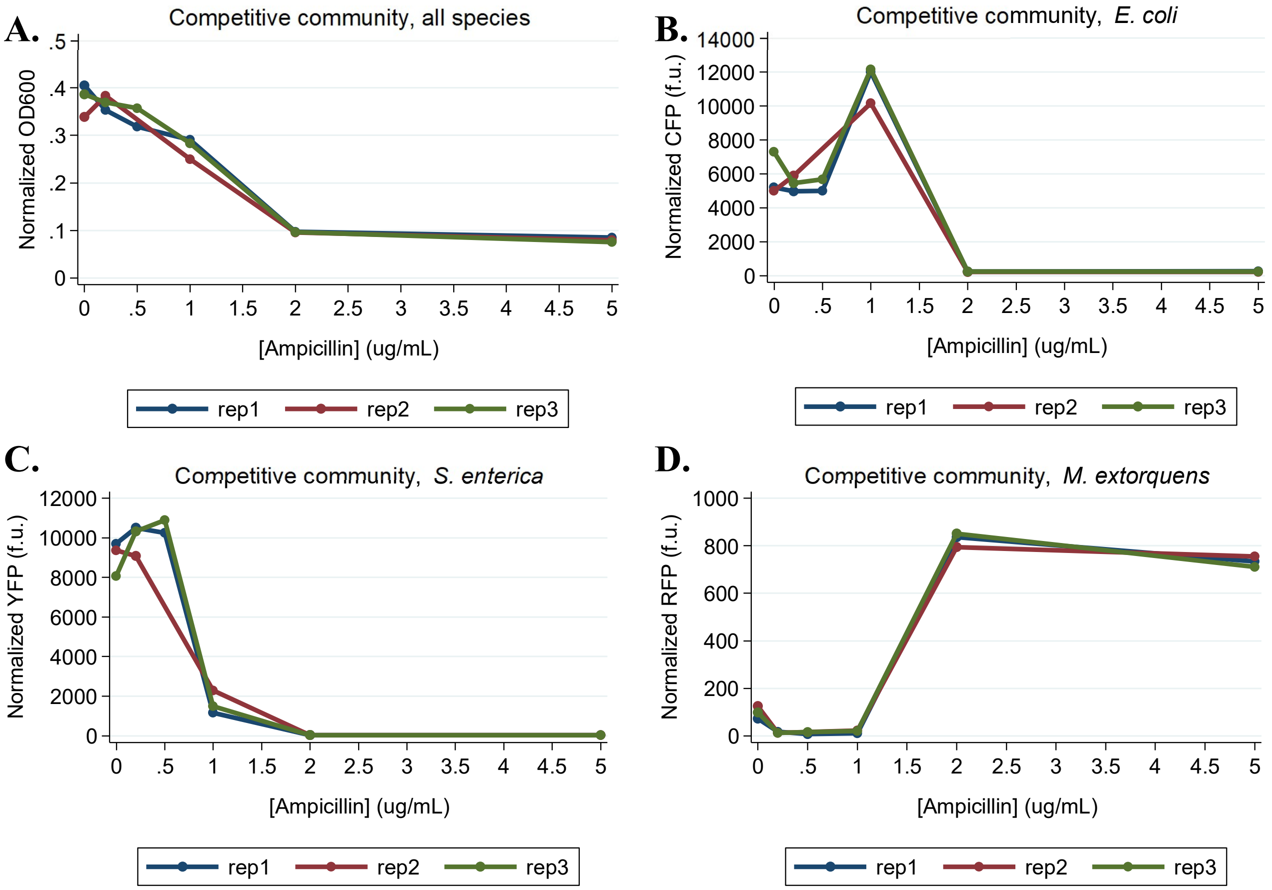

Supplementary figure 4. Competitive release of *M. extorquens* at high ampicillin concentrations in competitively grown community. Species were grown together in competitive Hypho medium at a range of ampicillin concentrations for 72 hours at 30°C. A Tecan InfinitePro 200 plate reader was then used to measure OD600 of the entire community and fluorescent markers corresponding to individual species. Normalized OD600 (**A**), cyan fluorescent protein to detect *E. coli* (**B**), yellow fluorescent protein to detect *S. enterica* (**C**), and red fluorescent protein to detect *M. extorquens* (**D**) were calculated by subtracting blank values from cell-free medium.

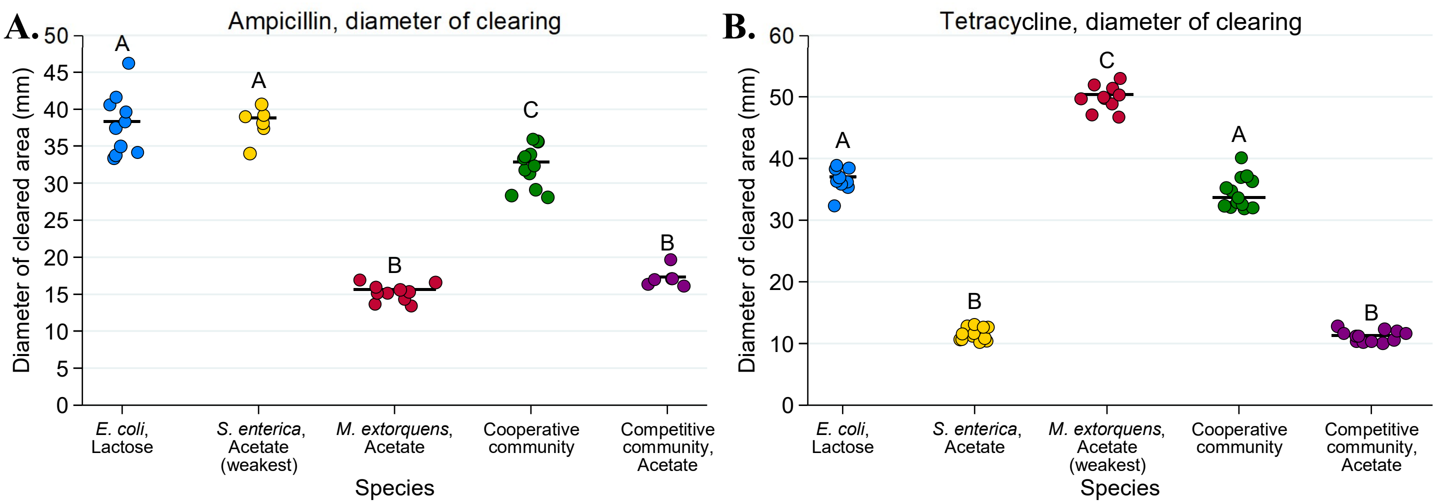

Supplementary figure 5. Acetate as the sole carbon source for *M. extorquens* and *S. enterica* in monoculture and competitive community on solid medium. Disc diffusion experiments using ampicillin (**A.**) and tetracycline (**B.**) were performed and analyzed as previously described. The 'weakest link' species (i.e. the species with the largest median zone of clearing in monoculture) is indicated on the x-axis. At least six replicates were performed for each community type. Pairwise comparisons of median zone of clearing were performed using a Mann-Whitney U test with Bonferroni adjustment for ten multiple comparisons. Matching letters represent statistical non-significance in medians between groups.

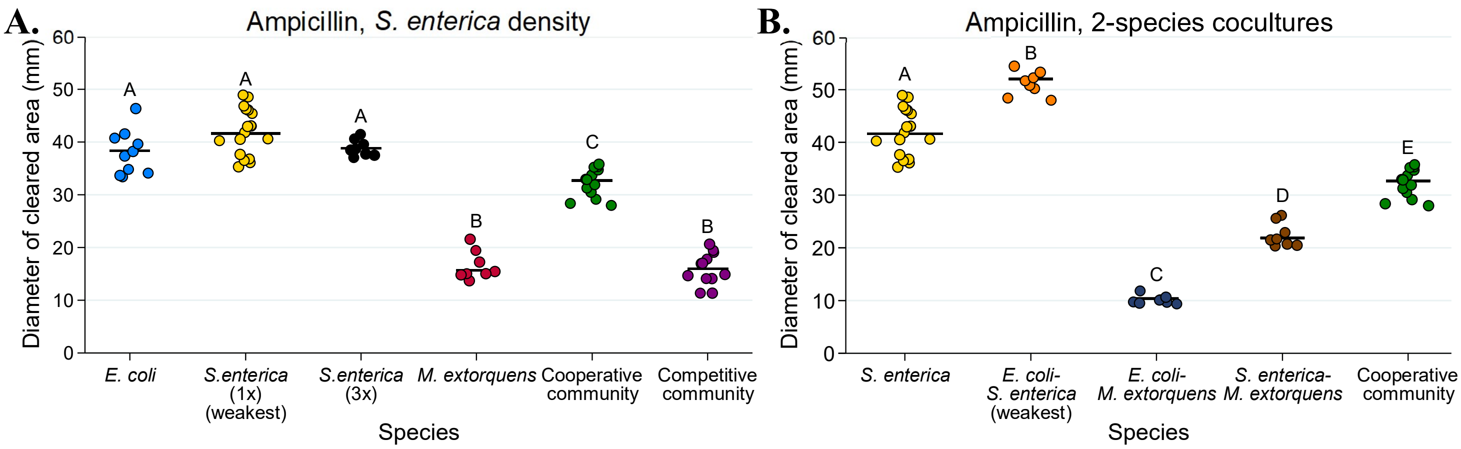

Supplementary figure 6. Possible mechanisms of *S. enterica* and *E. coli* protection from ampicillin in cooperative community on solid medium. The 'weakest link' species (i.e. the species with the largest median zone of clearing in monoculture) is indicated on the x-axis. **A.** Comparing monocultures and cooperative/competitive community zones of clearing to 3x *S. enterica* to match total cell density of *S. enterica* monoculture to total cell density in community. **B.** Two-species cooperative co-cultures as compared to *S. enterica* monoculture and cooperative three-species community. Pairwise comparisons of median zone of clearing were performed using a Mann-Whitney U test with Bonferroni adjustment for fifteen (**A.**) or ten (**B.**) multiple comparisons. Matching letters represent statistical non-significance in medians between groups.

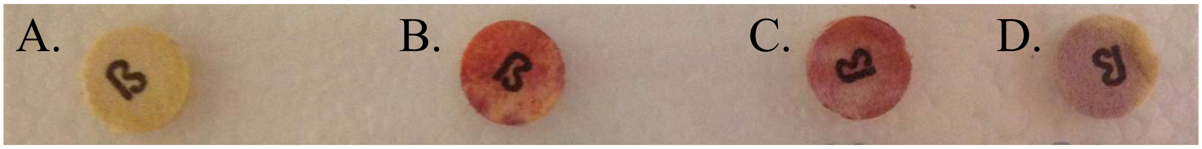

Supplementary figure 7. Nitrocefin disc assay for *M. extorquens*. Nitrocefin discs were used to determine if *M. extorquens* was producing a β-lactamase (indicated by a color change from yellow to red/pink). *E. coli* containing the plasmid pBR322 was used as a control; pBR322 contains a *bla* selectable marker encoding a β-lactamase. **A.** *M. extorquens* grown in liquid medium **B.** *M. extorquens* grown on solid medium **C.** *E. coli* with pBR322 on solid medium **D.** *E. coli* with pBR322 in liquid medium. For liquid cultures, 15uL of liquid medium containing cells was pipetted onto the disc and left at room temperature for 15 minutes. For solid cultures, colonies were scraped off plates and dissolved in liquid medium, and 15uL was pipetted onto the discs. All cells were grown in the presence of 50µg/mL ampicillin.

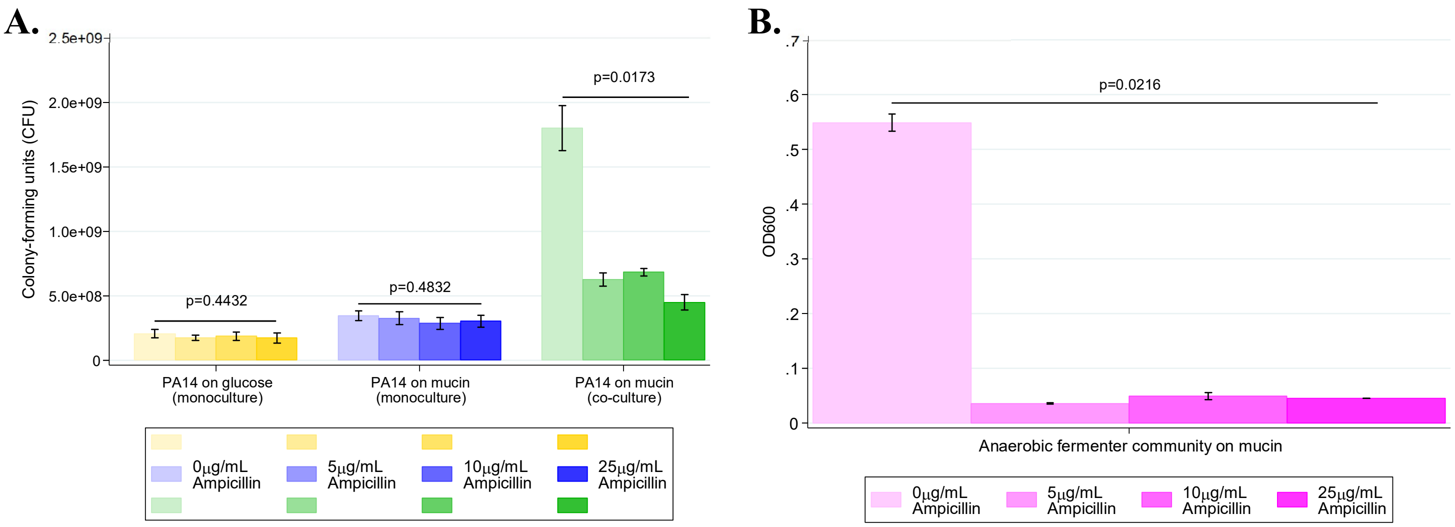

Supplementary Figure 8. Raw colony forming unit (CFU) data for *Pseudomonas aeruginosa* PA14 growth on mucin and glucose (**A**) and raw OD600 data for the anaerobic fermenter community on mucin (**B**). PA14 CFU grown alone on glucose and mucin, as well as in co-culture with the fermenter community on mucin. CFU values were obtained by plating cells from each concertation of ampicillin on Luria Broth agar. Note that CFU values in 0µg/mL ampicillin are higher on mucin than on glucose because of the greater amount of carbon available in 12mM of mucin vs. 12mM of glucose. OD600 values were obtained using a Biotek Synergy H1 plate reader. Each point represents the mean and standard deviation of triplicate samples. *P*-values were calculated using a Kruskal-Wallis test across ampicillin concentrations.

Supplementary table 1. Components of Hypho media.

Growth supported	Metabolites	Sulfate solution	Phosphate solution	Trace Metals
E. coli monoculture	2.78mM lactose, 0.020mM methionine	3.78mM $(NH_4)_2SO_4$, 0.814mM $MgSO_4$	14.5mM K_2HPO_4, 16.3mM NaH_2PO_4	1.2uM $ZnSO_4$<br>1uM $MnCl_2$<br>18uM $FeSO_4$<br>2uM $(NH_4)_6Mo_7O_{24}$<br>1uM $CuSO_4$<br>2mM $CoCl_2$<br>0.33um Na_2WO_4<br>20uM $CaCl_2$
S. enterica monoculture	5.55mM glucose			
S. enterica monoculture (acetate)	12.04mM acetate			
3-species competitive community	2.78mM lactose, 0.020mM methionine, 5.55mM glucose, 3.70mM succinate, 0.231mM methylamine			
3-species competitive community (acetate)	0.280mM lactose, 0.020mM methionine, 12.0mM acetate, 0.231mM methylamine			
E. coli- S. enterica cooperative co-culture	2.78mM lactose			
M. extorquens monoculture	3.70mM succinate, 0.231mM methylamine	3.78mM Na_2SO_4, 0.814mM $MgSO_4$		
M. extorquens monoculture, acetate	12.0mM acetate, 0.231mM methylamine			
3-species cooperative community	2.78mM lactose, 0.231mM methylamine			
E. coli- M. extorquens cooperative co-culture	2.78mM lactose, 0.020mM methionine, 0.231mM methylamine			
S. enterica- M. extorquens cooperative co-culture	5.55mM glucose, 3.70mM succinate, 0.231mM methylamine			

Supplementary table 2 Summary statistics for diameters of zones of clearing obtained by OD600 in ampicillin

Species	mean	std. dev	median	IQR
E. coli	38.06668	3.943439	38.33335	5.666698
S. enterica	42.09259	4.274579	41.66665	7.666698
M. extorquens	16.66668	2.488071	15.6667	2.6667
Cooperative community	32.35896	2.551192	32.6667	2.6667
Competitive community	15.98611	2.801933	16	3.41665

Supplementary table 3. Summary statistics for diameters of zones of clearing obtained by OD600 in tetracycline

Species	mean	std. dev	median	IQR
E. coli	36.66666	1.972022	37	1.333302
S. enterica	10.08333	0.8393739	9.916665	1
M. extorquens	37.63888	3.337767	36.8333	3.833349
Cooperative community	34.66668	2.400134	33.6667	4
Competitive community	8.944443	0.9525795	8.83333	2

Supplementary table 4. Summary statistics for diameters of zones of clearing obtained by fluorescence in ampicillin

Species	Community type	mean	std. dev	median	IQR
E. coli	monoculture	45.13889	1.709505	44.65	2.433334
E. coli	cooperative	39.7125	0.907629	39.9	1.316668
E. coli	competitive	46.07667	0.878418	46.26667	0.733334
S. enterica	monoculture	48.48333	0.314643	48.56667	0.433334
S. enterica	cooperative	43.03667	2.219301	42.75	1.333332
S. enterica	competitive	46.04	0.994155	46.36667	1.166664
M. extorquens	monoculture	25.86667	1.787612	26.55	2.566666
M. extorquens	cooperative	36.77667	3.72751	37.23333	2.633335
M. extorquens	competitive	24.12	1.820202	24.53333	1.700001

Supplementary table 5. Summary statistics for diameters of zones of clearing obtained by fluorescence in tetracycline

Species	Community type	mean	std. dev	median	IQR
E. coli	monoculture	40.17	1.094143	39.9	2.199997
E. coli	cooperative	40.37	1.607809	40.41667	1.933331
E. coli	competitive	38.84	1.091131	38.66667	0.966667
S. enterica	monoculture	8.733333	0.311111	8.666667	0.366667
S. enterica	cooperative	41.53667	1.334115	41.16667	1.266666
S. enterica	competitive	9.65	0.488447	9.85	0.733333
M. extorquens	monoculture	44.59	0.903772	44.43333	1.666668
M. extorquens	cooperative	31	1.428063	31.16667	2.399999
M. extorquens	competitive	.	.	.	.
